# The Natural Language Environment of 9-Month-Old Infants in Sweden and Concurrent Association With Early Language Development

**DOI:** 10.3389/fpsyg.2020.01981

**Published:** 2020-08-26

**Authors:** Sandra Nyberg, Mary Rudner, Ulrika Birberg Thornberg, Felix-Sebastian Koch, Rachel Barr, Mikael Heimann, Annette Sundqvist

**Affiliations:** ^1^ Infant and Child Lab, Department of Behavioural Sciences and Learning, Linköping University, Linköping, Sweden; ^2^ Linnaeus Centre HEAD, Department of Behavioural Sciences and Learning, Linköping University, Linköping, Sweden; ^3^ Department of Psychology, Georgetown University, Washington, DC, United States

**Keywords:** digital media, gesture, infant, language development, language environment, language ENvironment analysis, speech

## Abstract

The language environment is important for the development of early communication and language. In the current study, we describe the natural home language environment of 9-month-old infants in Sweden and its concurrent association with language development. Eighty-eight families took part in the study. The home language environment was measured using the Language ENvironment Analysis (LENA) system, and language development was assessed using Swedish Early Communicative Development Inventory (SECDI), a parent questionnaire. LENA measures showed dramatic variation between individuals but were comparable to and showed overlapping variance with previous studies conducted in English-speaking households. Nonetheless, there were significantly more infant vocalizations and conversational turns in the present study than in one previous study. Adult word count correlated significantly and positively with infants’ Use of gestures and the subscale of that section Communicative gestures. These together with another four non-significant associations formed a consistent overall pattern that suggested a link between infants’ language environment and language development. Although the direction of causality cannot be determined from the current data, future studies should examine children longitudinally to assess the directionality or the bidirectionality of the reported associations between infant’s language environment and language development.

## Introduction

The language environment of young children (i.e., all language surrounding the child) varies considerably, both quantitatively and qualitatively (e.g., Hart and Risley, 1995). The language environment is important for the development of early communication and language. In recent years, it has changed rapidly with increased access to, and use of, digital media. Technological tools like the Language ENvironment Analysis (LENA) system have dramatically improved the accuracy and ease of measurement of children’s language environment in their natural home settings (Schwarz et al., 2017). The purpose of the current study was to measure using LENA the natural home language environment of 9-month-old infants in Sweden and to investigate the concurrent association with language development for the first time in this age group. This was also the first report of 9-month-olds using LENA to be conducted in Sweden.

The preverbal infant communicates by using sounds and body language (Bishop, 1997). Infant vocalization begins to approximate the contours of syllables at around 3–4 months and becomes more frequent between 6 and 9 months as babbling increases (Gross, 2018). The use of gestures to communicate is viewed as the beginning of symbolic communication (Hoff, 2014). Gestural communication becomes more intentional at around 8–10 months; for instance, when the infant indicates a wish to be picked up by raising their arms or points at an unreachable object (Goldin-Meadow, 2006; Gross, 2018). Infants’ preverbal use of gestures has also been linked to subsequent language development. For example, gestural development at the age of 9 months has been shown to correlate with language production, both concurrently and predictively at 16 months (Sundqvist et al., 2016). Also, children raised in families with high socio-economic status (SES) have been shown to frequently use communicative gestures at 14 months. Further, differences in gestural use at 14 months could explain the fact that children from high SES backgrounds had larger vocabularies at 54 months (Rowe and Goldin-Meadow, 2009). Children engage in gestural communication and understand words before they produce their first word at around 12 months (Saxton, 2017; Gross, 2018), and therefore there is a need to distinguish between language comprehension and production especially in a study at 9 months.

Individual differences in early communication and language development are large (Fenson et al., 1994; Eriksson and Berglund, 1999). Early communication and language development can be assessed using the Swedish Early Communicative Development Inventories [SECDIs; the Swedish adaptation of the MacArthur-Bates Communicative Development Inventories (MB-CDIs)]. According to SECDI, the number of different words comprehended at 9 months typically ranges from 4 (20th percentile) to 27 (80th percentile; Eriksson and Berglund, 1999).

Exposure to adult speech is an important part of the infant’s language environment and varies considerably (Hart and Risley, 1995). For example, Hart and Risley (1995) found that infants in families with higher SES heard more individual word tokens from adults. They therefore emphasized the importance of future research taking SES and cultural background into consideration. Many studies have shown that the more words children hear, the more rapid their vocabulary acquisition (e.g., Hart and Risley, 1995; Hurtado et al., 2008; Weisleder and Fernald, 2013). This led to the belief that merely exposing infants to indiscriminate verbosity would close the socio-economic gap in language development. However, this is not the case (Hirsh-Pasek et al., 2015; Golinkoff et al., 2019). Recent studies have demonstrated that relationship between parent language input and child language outcomes is more dependent on quality than quantity. For example, Pan et al. (2005) found a positive relation between diversity of parental lexical input and infants’ subsequent vocabulary production in low-income families but not between parental talkativeness and early language development. Another study found both a concurrent and a predictive association between “parentese” parental speech and speech development but not between standard parental speech and infant speech (Ramírez-Esparza et al., 2014).

Apart from adult speech as such, interaction with adults is another important part of the infant’s language environment. Families differ in how much parents and children interact with each other (e.g., Zimmerman et al., 2009; Greenwood et al., 2011). Parent-infant interaction during the first year of life has been investigated with laboratory-based approaches (Gros-Louis et al., 2006), and recent advances like LENA can be used to examine these patterns in the natural language environment. For instance, Zimmerman et al. (2009) showed that the number of interactions between adults and young children aged 2–48 months is associated with concurrent language development and predicts subsequent language development, and suggested that interaction is more important for language development than quantity of adult speech. Further, children who experience more adult-child conversational turns (independently of SES, IQ, or adult speech exposure) display greater activation in the language production region in left inferior frontal cortex during language processing (Romeo et al., 2018). In fact, this greater activation mediated the relation between adult-child conversational turns and language skills in children aged 4–6 years (Romeo et al., 2018).

A newer element in infants’ language environment is digital media. Today’s infants are born into a digital media world, and many are exposed to digital media from a very young age (Chassiakos et al., 2016). The Swedish Media Council (2019) reported that in Sweden 54% of children under the age of 2 years used internet and 22% used apps. In the United States, the average child between 8 months and 8 years was found to be exposed to 232 min of background TV on a typical day (Lapierre et al., 2012). The question is whether exposure to digital media influences early language development. Some studies have linked early onset of digital media viewing and high frequency of viewing with delayed language development (e.g., Zimmerman et al., 2007; Chonchaiya and Pruksananonda, 2008), while other studies have drawn the conclusion that TV viewing during the first years of life had no effect on language development (Schmidt et al., 2009; Taylor et al., 2018).

Inconsistency in reports may be due to mediating factors. Specifically, researchers have emphasized the importance of adult-child interaction as the mediator between digital media and language development (e.g., Zimmerman et al., 2009; Mendelsohn et al., 2010; Masur et al., 2016). For example, for children aged between 2- and 48-months, TV viewing was a significant negative predictor of language development (Zimmerman et al., 2009). Further analysis indicated, however that the negative association between TV viewing and language outcomes was mediated by the frequency of adult-child interactions. Thus, it is important for parents to talk with their child and not only to their child (Zimmerman et al., 2009). Indeed, infants between 9 and 10 months showed phonetic learning from live interactions but not from digital media (Kuhl et al., 2003). Therefore, it seems to be the interactions between the child and the adult, rather than the amount of words in the vicinity of the child that are crucial for scaffolding the child’s developing language (Kuhl et al., 2003).

Tools to measure language environment have advanced from field notes to include a variety of options. In the pioneering study conducted by Hart and Risley (1995), the observers visited the families’ homes to make recordings and take notes, and thereafter transcribed the recordings manually. Another frequently used method has been to videotape a parent-child play situation and transcribe the recording using different software packages (e.g., Conventions of the Child Language Data Exchange System – CHAT). For example, Pan et al. (2005) videotaped mother-child interactions at home and utilized CHAT to transcribe both the verbal and nonverbal behaviors of the mother and child. Since 2008, researchers have begun to use the LENA device to record and automatically collate speech in the home environment. LENA is based on a small recording device that is securely placed in the front pocket of a waistcoat worn by the child (LENA Research Foundation, 2015). Recordings of up to 16 h can be made and automatically analyzed to quantify the child’s sound environment in different categories. These categories include adult word count (AWC), child vocalization count (CVC), conversational turn count (CTC), and electronic sound (ES). AWC is the number of adult word tokens spoken to and near the child during the recording, and CVC is the number of continuous speech segments (i.e., a speech interval preceded and followed by a pause greater than 300 ms) spoken by the child. CTC is the total number of conversational interactions between the child and an adult, where one speaker initiates and the other responds within 5 s. ES is the total time of exposure to TV, radio, and other electronic sounds during the recording (LENA Research Foundation, 2015). It is important to bear in mind that LENA cannot capture the visual language environment or the gestural interactions that characterize early language.

A small-scale study used 12 h LENA recordings to investigate the language environment of 30 North American infants aged 12–20 months, and found dramatic variation between individuals in AWC (from less than 100 words to over 36,000 with an average of over 13,000), while variation within individuals over repeated measures was small (Greenwood et al., 2011). AWC did not correlate significantly with a preschool language measure but both CVC and CTC did. ES was not reported by Greenwood et al. (2011).

A large-scale study (*N* = 329) from the United States used LENA to investigate natural language environment in typically developing children between 2 and 48 months (Gilkerson et al., 2017). The data were collected between 2006 and 2009, and it is of particular interest for the purposes of the current study that the study by Gilkerson et al. (2017) focused on 9-month-old infants. They found that during a 12 h recording period, the AWC for 9-month-olds was on average over 13,000. The CVC was over 1,000 utterances, and the CTC was almost 300. In all three cases, there was considerable variance. Unfortunately, ES was not reported by Gilkerson et al. (2017). The same dataset (but not restricted solely to 9-month-olds) showed that greater AWC and CTC were significantly associated with better language development, both concurrently and predictively 18 months later (Zimmerman et al., 2009). An even more recent study found that CTC at 18–24 months of age could predict language ability and cognitive outcomes 10 years later (Gilkerson et al., 2018). These results further highlight the importance of early language environment and early parental intervention programs when necessary for subsequent child development (Gilkerson et al., 2018).

Zimmerman et al. (2009) reported that more ES was significantly associated with poorer language development, but when CTC was controlled for, ES was no longer independently associated with language development (Zimmerman et al., 2009). Further, and from the same dataset, a mean ES of 1.3 h was reported along with an association between greater ES and lower AWC, CVC, and CTC for children aged 2–48 months was found (Christakis et al., 2009).

In the current study, we used LENA to investigate the natural home language environment of 9-month-old infants in Sweden and its concurrent association with language development. We focused on variation in LENA measures within a single age group instead of studying variation between different age groups. This establishes a baseline association which can act as a reference in the interpretation of longitudinal associations. At the age of 9 months, the infant is at an important stage in language development with understanding apparent but production only just starting to emerge. Thus, the influence of the language environment as such is likely to be qualitatively different at this age than when production emerges and reinforces the effect of the language environment. This study was conducted in a Swedish-speaking context, which helps to extend our empirical understanding of the early language environment and its association with language development across cultures. Specifically, 9-month-old infants in Sweden are typically still in the home environment, and thus language environment at this age is likely to have been stable over time. This means that concurrent associations between language environment and language development will reflect any earlier effect of language environment on language development.

The purpose of the current study was to measure using LENA the natural home language environment in 9-month-old infants in Sweden and to investigate the concurrent association with language development for the first time in this age group and country. On the basis of previous studies (Christakis et al., 2009; Zimmerman et al., 2009; Gilkerson et al., 2017), we predicted an average AWC of just over 13,000. We also predicted a CVC of just over 1,000 utterances and a CTC just under 300. Further, we predicted an average ES of just over 1 h. Finally, we expected that higher AWC, CVC, and CTC as well as lower ES would be associated with better language development, namely more Use of gestures and more Word comprehension.

## Materials and Methods

### Procedure

All infants included in the current study were a part of a larger longitudinal study. Recruitment to the longitudinal study was through the Swedish Population Register (SPAR). All families in a selected region of Sweden with an infant aged 9 months during one of two data collection periods in 2017 received an invitation to participate in the study. In total, 1,324 invitations were sent.

### Participants

Among those families who responded to the invitation to participate, 88 were included in the current dataset. This was after 13 were excluded, four of these declined to participate, and datasets for nine were lost due to data storage failure. The sample included 40 girls and 48 boys. Mean age on the LENA recording day was 9.51 months (*SD* = 0.26, Min = 9.04, Max = 10.22). Three of the participants were 10 months old. All but one of the participants were full-term (*M* = 40.6 weeks, *SD* = 1.37), and parents reported normal Apgar score at 5 min for all but one of the participants. Mean birth weight was 3,564 g (*SD* = 470 g), and mean birth length was 50.9 cm (*SD* = 2.02 cm). All parents reported that their infant was typically developing and had no known medical issues. Among the participants, 53 were the only child in their family, while 26 had one older sibling and nine had two. None of the participants had begun childcare. All families reported Swedish as main language at home (79 families reported that both parents speak Swedish, and nine families reported that one parent speaks another language). The families were highly educated; and 83% of mothers and 66% of fathers had a university degree.

### Measures

#### Language Environment

LENA was used to record the participants’ language environment. Parents were instructed to choose a typical day and dress their infant with the LENA vest during the morning routine and wear it during the whole day (except at bath time). Parents were given the option of deleting the recorded data afterward if they felt it compromised their privacy (without the research team listening), but no parent chose to delete data. The collected audio recordings were downloaded into, analyzed in, and extracted from the LENA software advanced data extractor (ADEX). ADEX categorizes the data into different variables and time intervals (LENA Research Foundation, 2011). The minimum recommended time interval is 10–12 h to get the most reliable recordings analysis (LENA Research Foundation, 2015). In the current study, 85 recordings were at least 12 h. The portion exceeding 12 h was cut from these recordings. The remaining three recordings were 11.1, 11.2, and 11.3 h long. These recordings did not differ from the other 85 in the proportions of different types of sound. The included variables in the current study are AWC, CVC, CTC, and ES.

#### Language Development

Language development was measured through the SECDIs-words and gestures (SECDI-w&g), which is a Swedish adaptation of the established and well-validated MB-CDIs (Fenson et al., 1994; Eriksson and Berglund, 1999). The SECDI-w&g is an index of communicative skills in children aged 8–16 months based on parental reports (Eriksson and Berglund, 2002). It is divided into three main sections of early communicative development: Word comprehension, Word production, and Use of gestures. Word comprehension and Word production were calculated by adding the number of different items the parent had marked from a vocabulary checklist consisting of 382 items. Use of gestures included 62 items. Fifty-one of these items had a binary response (yes–no, scored as 1–0) and were scored according to the manual (Eriksson and Berglund, 1999; Fenson et al., 2007). The other 11 items had a three-point rating scale (not yet–sometimes–often, scored as 0–1–2), and so to capture more variance, we scored these items in line with previous studies (e.g., Sundqvist et al., 2016) instead of using the binary coding prescribed by the manual. This means that Use of gestures took into account both how many different gestures the infant used and also to some extent how often the infant used the gesture. The section Use of gestures consists of five subscales: Communicative gestures, Games and routines, Action with objects, Pretend to be a parent, and Imitating other adult actions.

We decided *a priori* that if we found a statistically significant correlation between any of the LENA variables and Use of gestures, we would also perform an explorative analysis of the correlation between that LENA variable and the Use of gestures’ subscale Communicative gestures. This subscale has been shown to provide valid data during the first year of life and to be strongly predictive of the emergence of meaningful speech (Fenson et al., 2007). The maximum score was 382 for both Word comprehension and Word production. The maximum score for Use of gestures was 73, and the subscale Communicative gestures which consists of the 11 items that have a three-point rating scale has a maximum score of 22. SECDI was filled in by a parent and administered online.

#### Data Analysis

We computed mean values and standard deviations for the four LENA variables and the three SECDI sections. LENA mean values were compared to published data using independent samples *t*-tests. In the current dataset, Pearson’s correlations were computed among the LENA variables, among the SECDI sections and between the LENA variables and the SECDI sections and one subscale (Communicative gestures). As regards the specific directional correlation predictions, we computed one-tailed correlations. Non-predicted correlations were tested using two-tailed tests.

## Results

### Language Environment

The 88 9-month-old infants enrolled in the current study heard an average of 15,152 AWC during the 12 h LENA recording in a natural home setting (see Table 1). One infant heard 2,720 adult words while another infant heard over 13 times more during the same time period – 37,599 words. The average recorded CVC was 1,267, and the infant with highest number of vocalizations (2,722) showed almost 10 times more vocalizations compared to the infant that had the lowest number of vocalizations (285). The average CTC was 333, and between families the conversational turns varied from 64 to 776 – thus over 12 times more turns for the family with the highest CTCs. On average, the infants were around ES for 15:26 min (*SD* = 18:37). However, two infants were around ES for 1 h or more, and indeed, median ES was only 10:02 min. There was no evidence that number of siblings, sex, or SES influenced any of the LENA variables.

**Table 1:** Language ENvironment Analysis (LENA) variables of interest, mean, standard deviation, Min, Max, and Pearson’s correlations.

Variable	*M*	*SD*	Min-Max	1	2	3
1. Adult word count	15,152	6,791	2,720–37,599			
2. Child vocalization count	1,267	496	285–2,722	0.151		
3. Conversational turn count	333	135	64–776	0.597^***^	0.758^***^	
4. Electronic sound (hh:mm:ss)	00:15:26	00:18:37	00:00:21–01:51:15	−0.030	−0.036	−0.125

Pearson’s correlations are two-tailed and uncorrected for multiple comparisons; *N* = 88.

^***^p < 0.001.

**Figure 1:**
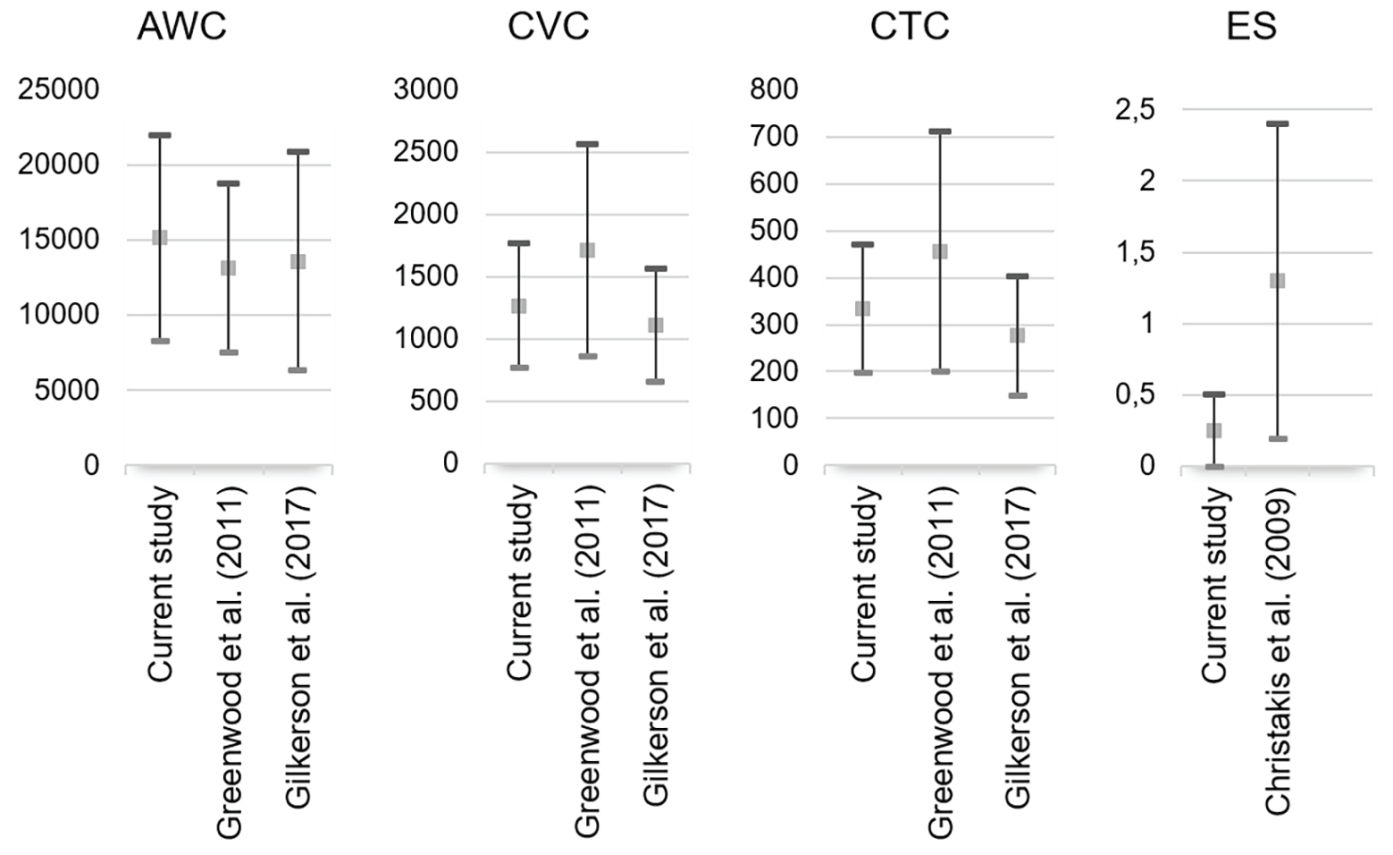
Language ENvironment Analysis (LENA) variables compared with previous studies. The square illustrates the mean, and the whiskers illustrate the standard deviations. AWC, adult word count; CVC, child vocalization count; CTC, conversational turn count; ES, electronic sound. Note that the scale on the y-axis differs between variables.

As shown in Table 1, parametric correlations within LENA variables revealed a significant correlation between AWC and CTC (*p* < 0.001) and between CVC and CTC (*p* < 0.001). Figure 1 shows LENA variables of interest in the current Swedish study in relation to similar variables in previous American studies and shows a similar pattern of results. In comparison to the study conducted by Gilkerson et al. (2017), our study showed significantly more CVC, *t*_(134)_ = 2.02, *p* = 0.045, standardized effect size = 0.33, and CTC, *t*_(133)_ = 2.63, *p* = 0.010, standardized effect size = 0.43, but no significant difference regarding AWC, *t*_(122)_ = 1.33, *p* = 0.185, standardized effect size = 0.23. It was not possible to make a similar statistical comparison with any previous study for the ES variable (e.g., Christakis et al., 2009) because data for 9-month-old infants was not reported separately.

### Language Development

Of the 88 families that recorded a typical day, 78 also filled in the SECDI (34 girls and 44 boys, 11.4% internal attrition). The average number of words comprehended for Word comprehension section was 22.08 (*SD* = 23.37, Min = 0, Max = 119), and the average number of words produced for the Word production section was 0.62 (*SD* = 1.22, Min = 0, Max = 6). The average score for Use of gestures was 11.60 (*SD* = 5.60, Min = 3, Max = 26). Within the section Use of gestures, the average score on the subscale Communicative gestures was 6.18 (*SD* = 3.29, Min = 0, Max = 13). These results are in line with previous work (Sundqvist et al., 2016). There was no evidence that number of siblings or sex influenced SECDI scores.

Regarding the different sections in SECDI, there was a significant Pearson’s correlation between Word comprehension and Use of gestures (*r* = 0.37, *p* = 0.001) and between Word comprehension and the subscale named Communicative gestures (*r* = 0.31, *p* = 0.005). There was also a correlation between the section Use of gestures and the subscale Communicative gestures (*r* = 0.80, *p* < 0.001). These correlations were two-tailed and uncorrected for multiple comparisons.

### Language Environment and Language Development

**Figure 2:**
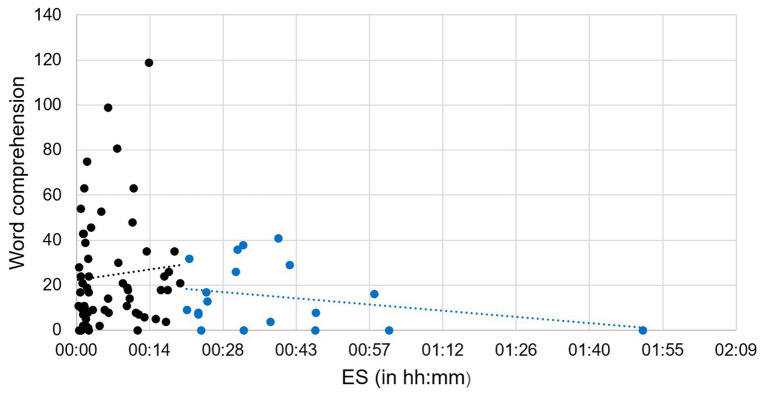
Scatterplot (*n* = 78) of the non-significant negative association between ES and Word comprehension. Black dots indicate participants with ES less than 20 minutes and blue dots indicate participants with ES greater than 20 minutes.

As shown in Table 2, the concurrent correlation between LENA and SECDI demonstrated a statistically significant association between AWC and Use of gestures (*r* = 0.24, *p* = 0.018). There were no other statistically significant correlations between LENA variables and SECDI section scores. However, close inspection of Table 2 reveals a pattern of non-significant associations (*ps* ≤ 0.10) between SECDI scores for the section Word comprehension and three of the LENA variables. This pattern is in line with our predictions. Specifically, there were *positive*, albeit non-significant, correlations with AWC (*p* = 0.064) and CTC (*p* = 0.099) as well as a *negative*, albeit non-significant, correlation with ES (*p* = 0.081). Further, there was a non-significant positive correlation between Use of gestures and CTC (*p* = 0.065), and was also in line with our predictions. Inspection of the scatterplot in Figure 2 illustrating the non-significant correlation between Word comprehension and ES suggested a possible pattern of two different subgroups, such that participants with ES < 20 min might comprehend more words than participants with ES > 20 min. However, an independent samples *t*-test showed no significant difference in Word comprehension between these two subgroups. Inspection of scatterplots (not reproduced here) of the other non-significant associations that were consistent with our predictions provided no evidence of any kind of systematic relation other than that indicated by the non-significant correlation. Because of multicollinearity and the lack of statistical significance for all correlations except one, regression analyses could not be performed.

**Table 2:** Coefficients of the Pearson’s intercorrelations between LENA and Swedish Early Communicative Development Inventory (SECDI).

	SECDI		
LENA	Word comprehension	Word production	Use of gestures
Adult word count	0.17^†^	0.04	0.24^*^
Child vocalization count	0.06	−0.03	−0.05
Conversational turn count	0.15^†^	0.11	0.17^†^
Electronic sound	−0.16^†^	−0.06	0.01

^†^*p* ≤ 0.10;

^*^p < 0.05.

*n* = 78.

**Figure 3:**
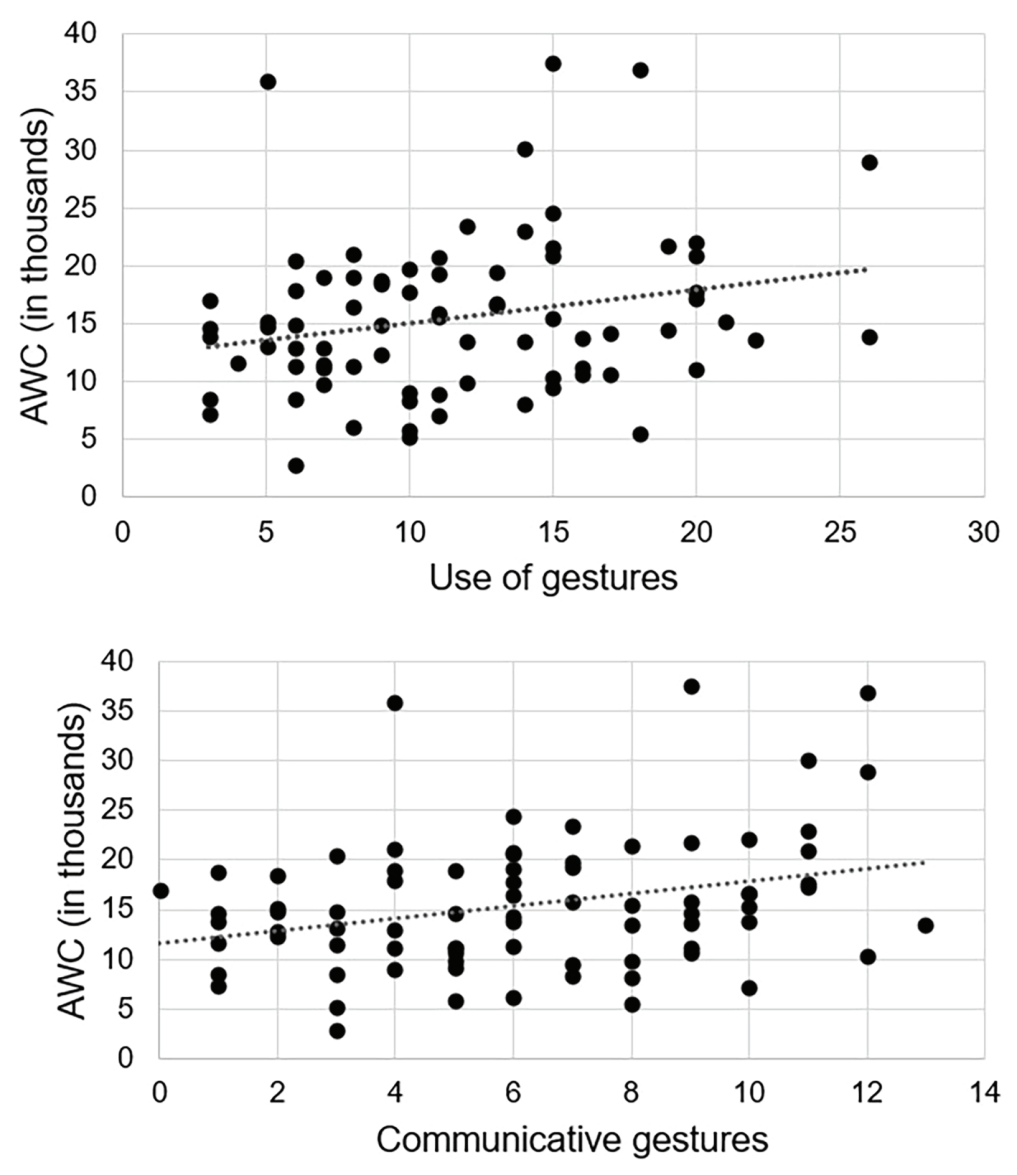
Scatterplots (*n* = 78) of the statistically significant associations between the LENA variable adult word count and the SECDI section Use of gestures **(upper panel)** and Use of gestures subscale Communicative gestures **(lower panel)**.

Since the correlation between the LENA variable AWC and the SECDI section Use of gestures was statistically significant, we further explored the association between AWC and the Use of gestures subscale Communicative gestures. The correlation between AWC and Communicative gestures was also statistically significant (*r* = 0.30, *p* = 0.008; see Figure 3).

## Discussion

In the current study, we describe the natural home language environment in a sample of 9-month-old infants from Swedish households and relate it to concurrent language development.

### Language Environment

As expected, AWC, CVC, and CTC in the current study were all of the same order of magnitude as in previous studies by Greenwood et al. (2011) and Gilkerson et al. (2017) with overlapping variance between studies as shown in Figure 1. Notwithstanding, statistical tests showed that CVC and CTC were significantly higher in the present study than in the study by Gilkerson et al. (2017), although there was no significant difference in AWC.

To date, to our best knowledge, only Gilkerson et al. (2017) has used LENA as a data collection method to describe language environment at specifically 9 months of age. LENA was originally developed for American English and has so far been found to be reliable (albeit slightly less so than for American English) for Spanish, Mandarin, and French (Weisleder and Fernald, 2013; Gilkerson et al., 2015; Canault et al., 2016). In Swedish, only the variable AWC has previously been evaluated, and showed reliability similar to that of other non-English studies (Schwarz et al., 2017). Thus, it is likely that the lack of difference in AWC in the current study compared to Gilkerson et al. (2017) is also reliable and indicates that the parents of 9-month-old infants in Sweden talk as much as their American counterparts. Unfortunately, there is no previous study addressing the reliability of CVC and CTC data collected in Sweden using LENA. Therefore, we must be cautious in interpreting the larger number of child vocalizations and conversational turns identified in the current study compared to previous work. It may be the case that Swedish 9-month-old infants are more verbal than their American peers. One reason for this could be that Swedish social security legislation allows parents to spend at least 1 year at home with their newborns. However, we cannot rule out that differences are simply a measurement error (e.g., a possible lack of reliability regarding CVC and CTC in Swedish) or a reflection of fundamental linguistic differences. Further research should investigate this issue.

The average exposure to ES, according to LENA data, was 15 min (median 10 min) in the present study, thus substantially less than we hypothesized (i.e., just over an hour, based on Christakis et al., 2009). However, the range of data overlaps between the two studies, and thus it is not clear that they differ significantly. Unfortunately, we were unable to test the difference statistically as age ranges differ between studies. Future cross-cultural studies making direct comparisons across countries are necessary to test for potential differences in ES. Previous studies have shown that LENA underestimates ES (e.g., Zimmerman et al., 2009; Gilkerson et al., 2015), and technological advances making the sounds produced by digital media more lifelike are likely to further challenge the reliability of LENA. What is more, LENA only captures sounds from digital media and not overall digital media use (e.g., viewing Facebook, reading blogs or other silent activities, or personal viewing on mobile devices, where the volume may not be detected), and thus provides an underestimation of the latter. Our results also indicate a lower degree of exposure to ES than official figures, which reported that 15% of children under the age of 2 years in Sweden listen to music for 1–2 h per day and 11% watch movies or TV programs for 1–2 h per day (Swedish Media Council, 2019). Given public debate concerning the effect of digital media on child development, it is possible that the highly educated parents of infants participating in the study carefully control their infant’s excess to digital media or even deliberately turned it off during LENA recording. However, most parents in the current study indicated that they did not change their behavior or their digital media use on the recording day.

We found considerable variation between individuals in AWC, as well as CVC, CTC, and ES. For instance, AWC ranged from 2,720 words to 37,599, CTC ranged from 64 turns to 776, and ES ranged from 21 s to 1 h and 51 min. This is in line with previous studies, which have also shown dramatic variation between individual language environments measured with LENA, but previous studies have focused mainly on age-related patterns (e.g., Greenwood et al., 2011; Gilkerson et al., 2017) and not on within-age group variations.

Differences in language environment have previously often been associated with SES (e.g., Hart and Risley, 1995; Romeo et al., 2018). The sample in our study was homogeneous, and the participants belonged to families whose educational level was above average in Sweden (Statistiska centralbyrån, 2018). Therefore, based on SES, our sample is not representative, but it is noteworthy that we found major differences in language environment, although the educational level was high. Future studies should target a more representative cross-section of the population.

### Language Environment and Language Development

Our hypotheses that higher AWC, CVC, and CTC and lower ES would be associated with better concurrent language development as measured by SECDI were partly supported. The only statistically significant correlation between a LENA variable and a SECDI section was a positive correlation between AWC and Use of gestures. Subsequent explorative investigation of the association between AWC and the Use of gestures subscale Communicative gestures also showed a statistically significant positive correlation. Although none of the other correlations between LENA variables and SECDI sections were statistically significant, there was an overall pattern of correlations between infant Word comprehension as measured by SECDI and three of the LENA measures (AWC, CTC, and ES). While AWC and CTC were positively associated with Word comprehension, ES was negatively associated with Word comprehension. Further, CTC was positively associated with Use of gestures. Although these findings did not meet conventional levels of statistical significance, this overall pattern of results was in line with our hypotheses. Thus, although we only have strong evidence that adult talk is associated with language development in infants aged 9 months, it would be wrong to ignore the indication that infant Word comprehension is also linked to factors in the child’s language environment.

Previous studies have not studied the relation between LENA variables and language development at specifically 9 months. But, Greenwood et al. (2011) found that among infants who were 12–20 months old, the variable CTC, but not AWC, correlated positively with language development. Zimmerman et al. (2009) found that at 2–48 months old both AWC and CTC correlated positively with language development and ES correlated negatively, but AWC was partially mediated by CTC, and ES was fully mediated by CTC. Even though these studies included 9-month-old infants, the age range was considerably broader (with a mean age of 15.6 months) than in the current study. Thus, it is to be expected that their language skills were more developed than those of the participants in the current study and more likely to correlate with language environment measures. Further, it should be noted that the measure of language development used in the studies by Greenwood et al. (2011) and Zimmerman et al. (2009) differed from that used in the present study.

What the correlation between AWC and Use of gestures means is unclear. One probable explanation is that adult speech facilitates early communicative behavior, but it can also be in the opposite direction – that infants’ use of gestures itself encourages adults to speak more. The most reasonable explanation is probably that the relationship is bidirectional (cf. Rowe and Goldin-Meadow, 2009). Future work investigating this relationship longitudinally is ongoing.

We expected a significant correlation between CTC and certain aspects of language development, namely Word comprehension and Use of gestures, but we only found non-significant associations (*p* = 0.099, *p* = 0.065, respectively). One aspect to be aware of is that LENA can only capture verbal interactions and that 9-months-old infants often use non-verbal communication to interact with others, for example, eye-contact, pointing, or raising their arms. Even though we do not have a broad picture of the adult-infant interaction, we can see non-significant tendencies toward more adult-infant interactions and better language development. With that said, it may still be the case as Zimmerman et al. (2009) suggested, that adult-infant interactions are at least as important as adult words. Future research could directly measure parent-infant gestural interaction patterns and assess in addition to LENA information, whether additional variance from these interactions are associated with infant SECDI language outcomes.

A non-significant tendency toward a negative correlation between ES and Word comprehension was observed (*p* = 0.081). Our sample was exposed to appreciably less ES than expected and the lack of significant associations might be a reflection of the fact that low levels of ES and language are not in fact related at the age of 9 months. That is, a threshold of exposure may be needed for interference with early communication to occur. It might also be the case that our measurement was simply not sensitive enough to detect the small variations our sample displayed. Additional data cross-validating the LENA, ES with other measures across a larger and more diverse sample is needed to address these questions.

As expected, none of the LENA variables correlated with concurrent Word production. Results confirmed that the 9-month-old infants in the current study are largely preverbal and only a few had started speaking. Thus, variance in Word production was too limited to assess associations.

## Conclusion

This manuscript provides the first description of the natural home language environment according to LENA in a sample of 9-month-old infants in Sweden. Both the amount of AWC, CVC, and CTC and the intra-individual variation were comparable to and showed overlapping variance with previous studies conducted in English-speaking households. Despite this, we identified significantly more CVC and CTC than in Gilkerson et al. (2017). AWC correlated significantly and positively with concurrent SECDI Use of gestures and the subscale of that section Communicative gestures. We could also discern an overall pattern of four non-significant associations (*ps* ≤ 0.10) in the expected direction that together with the significant association might be interpreted as a link between infants’ language environment and language development. The direction of causality cannot be determined from the current data. Future studies should track associations between infant’s language environment and language development longitudinally to better assess directionality or bidirectionality of the associations.

## Data Availability Statement

The raw data supporting the conclusions of this manuscript are available on OSF at the following link https://osf.io/c4vtd.

## Ethics Statement

The studies involving human participants were reviewed and approved by the Regional Ethical Board in Linköping, Sweden (2016/490-31). Written informed consent to participate in this study was provided by the participants’ legal guardian/next of kin.

## Author Contributions

The study was conceived by MH, AS, RB, and F-SK. Data were collected by MH, AS, F-SK, and UB. Data were analyzed by F-SK and SN. The first draft of the manuscript was written by SN. MH, AS, F-SK, UBT, RB, SN, and MR contributed to the final version. All authors contributed to the article and approved the submitted version.

### Conflict of Interest

The authors declare that the research was conducted in the absence of any commercial or financial relationships that could be construed as a potential conflict of interest.
